# Advertising of tobacco and related products on social media in Germany

**DOI:** 10.18332/tpc/195499

**Published:** 2024-11-22

**Authors:** Christopher Heidt, Amelie Wüllner, Jana Seiler, Nobila Ouédraogo, Katrin Schaller

**Affiliations:** 1 German Cancer Research Center, Heidelberg, Germany

**Keywords:** social media, advertising, electronic cigarettes, heated tobacco products, germany

**Dear Editor,**

Advertising of tobacco and related products increases smoking initiation as well as the desire to try the products^1,2^. To protect adolescents from harmful products, advertising of tobacco and related products is banned on TV, radio, print, and the internet, including social media in Germany^3^. However, the law is poorly enforced on social media. As many adolescents use social media extensively^4^, they could be exposed to advertising of heated tobacco products (HTPs) as well as electronic cigarettes (e-cigarettes), and be encouraged to use these products. In 2023, we monitored the extent of advertising of e-cigarettes and HTPs on social media in Germany. From February to September 2023, we collected posts in the German language on Facebook, Instagram, TikTok, and Pinterest related to HTPs and e-cigarettes using a social media listening tool (Meltwater). The posts were systematically categorized by two coders and analyzed with regard to criteria such as the posting account and the subject of the post. In the period of February and March 2023, we discovered 756 promotional posts on e-cigarettes in Germany and 432 promotional posts on HTPs in the period of February to September 2023. Especially retailers (n=394; 52% of e-cigarette posts; and n=243; 56% of HTPs posts) and manufacturers (n=106; 14% of e-cigarette posts; and n=155; 36% of HTPs posts) are using social media to promote their products. The analysis creates a picture of how these products are advertised on social media: the promotional posts for HTPs often highlight technical features of the products (n=89; 21% of HTPs posts). The products are also presented as stylish and trendy accessories (n=91; 21% of HTPs posts), and in some cases, a young target group is addressed (n=147; 34% of HTPs posts). The promotional posts for e-cigarettes tend to emphasize the taste of the products (n=201; 27% of e-cigarette posts) and are largely about disposable products (n=289; 38% of e-cigarette posts), which are made attractive to adolescents by bright colors and various flavors. Our analysis shows for the first time the extent of advertising of e-cigarettes and HTPs on social media in Germany. Despite the ban on advertising and the self-regulation of the platforms, young users of the platforms are still exposed to advertising of harmful products. The data reveal existing regulatory weaknesses and will be incorporated into an advocacy campaign to drive forward legislation for a comprehensive ban on tobacco and alcohol advertising in Germany. At the same time, the results are a call for strict control of the existing regulation.

**Figure 1:**
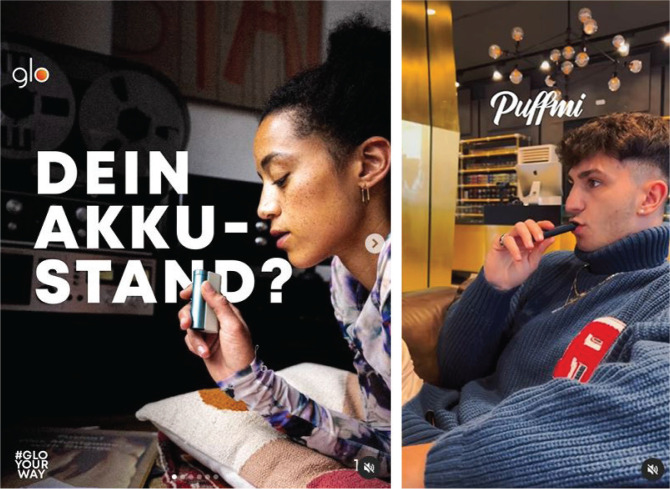
Manufactures promote HTPs and e-cigarettes on Instagram in Germany (the left post asks, ‘Your battery level?’)

## Data Availability

The data supporting this research are available from the authors on reasonable request.
